# Supplementary material for: Bypassing pan-enterovirus host factor PLA2G16
Source: Nat Commun. 2019 Jul 18;10:3171. doi: 10.1038/s41467-019-11256-z (PMC6639302; doi:10.1038/s41467-019-11256-z)
Supplement: Supplementary file 1 — Supplementary information [file 41467_2019_11256_MOESM1_ESM.pdf]

Supplementary Information

Bypassing pan-enterovirus host factor PLA2G16

Baggen et. al

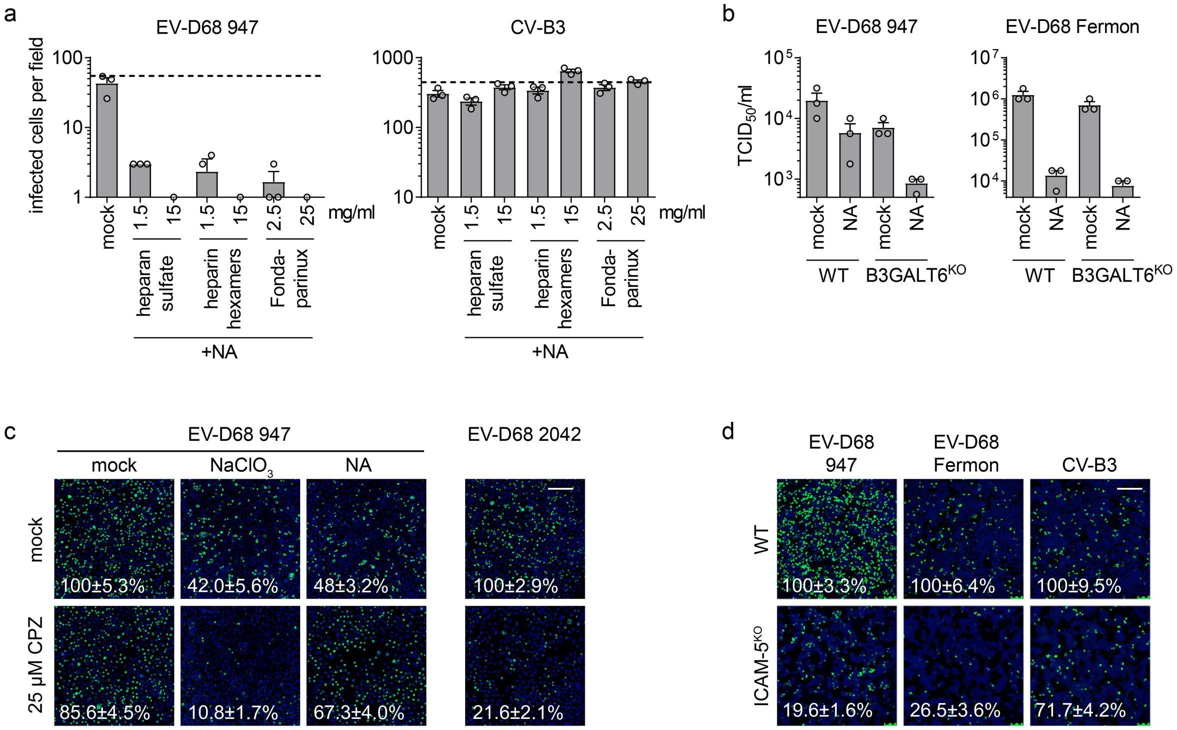

Supplementary Figure 1. EV-D68-947 employs sialic acid, sGAGs, and ICAM-5 as receptors. a, EV-D68-947 or coxsackievirus B3 (CV-B3) were incubated with different concentrations of the sGAG analogues heparan sulfate, heparin-derived hexasaccharide (dp6), or sulfated pentasaccharide (fondaparinux), followed by infection of neuraminidase (NA)-treated HeLa-R19 cells, dsRNA staining and quantification of infected cells. **b,** NA-treated WT or B3GALT6^{KO} HAP1 cells were infected with EV-D68 and yields of infectious virus (TCID50: median tissue culture infective dose) were determined after a single replication cycle. Error bars (a,b) represent the mean ± s.e.m. of 3 technical replicates. **c,** H1-HeLa cells were treated with neuraminidase (NA), sodium chlorate ($NaClO_3$) or chlorpromazine (CPZ) and infected with EV-D68, followed by staining of dsRNA (green) and nuclei (blue). Shown are representative confocal micrographs. Values denote the number of infected cells (mean ± s.e.m. of 4 technical replicates) as percentage of mock. The experiment was conducted twice with similar results. **d,** WT or ICAM-5^{KO} HAP1 cells were infected with EV-D68 or CAR-binding CV-B3, followed by staining of dsRNA (green) and nuclei (blue). Shown are representative confocal micrographs. Values denote the number of infected cells (mean ± s.e.m. of 4 technical replicates) as percentage of WT. The experiment was conducted twice with similar results. Scalebars represent 150 µm.

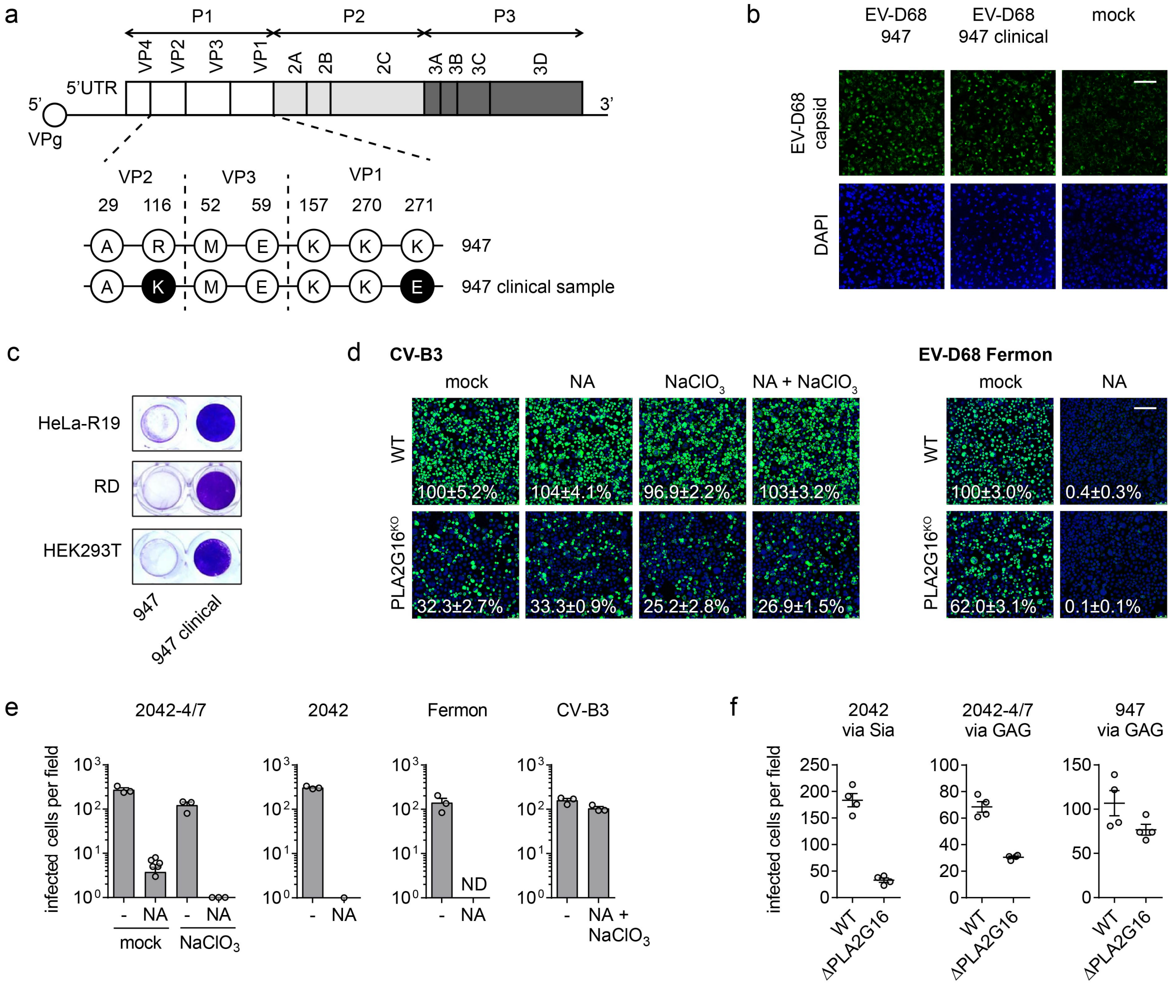

Supplementary Figure 2. a, Top, schematic representation of the enterovirus genome. Bottom, circles represent capsid residues that differ between EV-D68-947 and EV-D68-2042, as was previously described. Black circles indicate positions at which EV-D68-947 differs from the viral genomic sequence obtained from patient material. **b,** Huh7/lunet/T7 cells were transfected with plasmids encoding the genome of EV-D68-947 clinical, which matches the sequence found in patient material, or EV-D68-947, followed by staining of capsid proteins (green) and nuclei (blue). **c**, Lysate of transfected cells (b) was used to infect different cell lines, after which live cells were stained with crystal violet at 7 days postinfection. **d,** WT or PLA2G16^{KO} H1-HeLa cells were treated with neuraminidase (NA), sodium chlorate ($NaClO_3$) or a combination of both and infected with CV-B3 or EV-D68-Fermon, followed by staining of dsRNA (green) and nuclei (blue). Shown are representative confocal micrographs. Values denote the number of infected cells (mean ± s.e.m. of 3 technical replicates) as percentage of mock. Scalebars represent 150 µm. **e,** H1-HeLa cells were infected and stained for dsRNA, followed by quantification of infected cells. **f**, Raw data used to calculate values presented in Fig. 2e. Error bars represent the mean ± s.e.m. of ≥3 (e) or 4 (f) technical replicates.

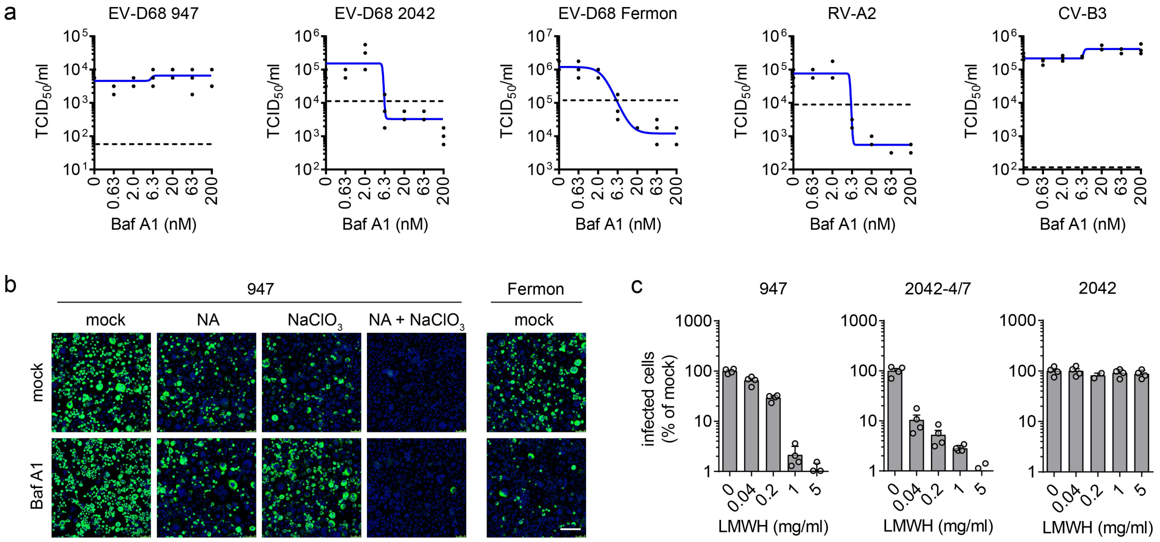

Supplementary Figure 3. a, Bafilomycin A1 (BafA1)-treated HAP1 cells were infected with EV-D68 strains, rhinovirus A2 (RV-A2) or coxsackievirus B3 (CV-B3) and yields of infectious virus (TCID50: median tissue culture infective dose) were determined after a single replication cycle. Three technical replicates with a fitted sigmoidal curve are shown. The experiment was conducted twice with similar results. **b,** H1-HeLa cells were treated with neuraminidase (NA), sodium chlorate ($NaClO_3$), 200 nM BafA1, or a combination and infected, followed by staining of dsRNA (green) and nuclei (blue). Shown are representative confocal micrographs. The scalebar represents 150 µm. The experiment was conducted twice with similar results. **c,** Viruses were incubated with low-molecular-weight-heparin (LMWH), followed by infection of HeLa-R19 cells, dsRNA staining and quantification of infected cells. Error bars represent the mean ± s.e.m. of 2 biological and 2 technical replicates.

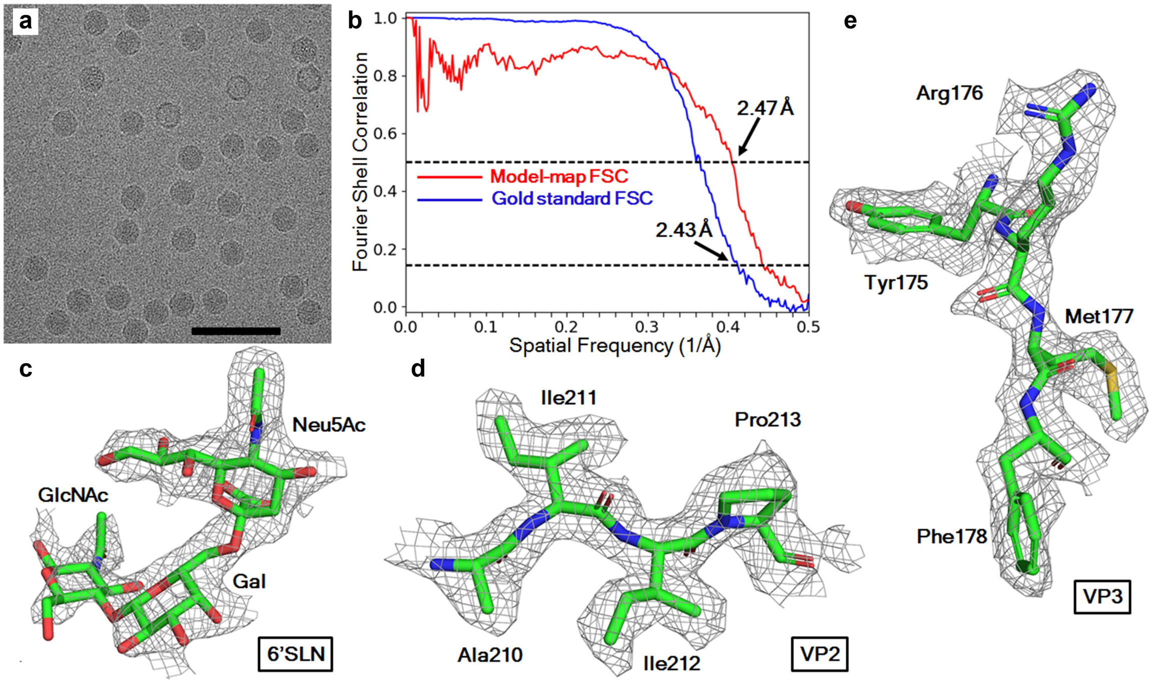

Supplementary Figure 4. The 2.4 Å resolution structure of EV-D68-947 in complex with 6'-sialyl-N-acetyllactosamine (6'SLN). **a,** A typical electron micrograph of the virus after incubation with 10 mg mL^{-1} 6'SLN at 33°C for about 30min. Scale bar: 100 nm. **b,** Fourier shell correlation (FSC) curves. Gold standard FSC is the FSC between two independently reconstructed half maps (FSC cutoff = 0.143). The "model-map" FSC is the FSC between the cryo-EM map and a map based on the atomic model (FSC cutoff = 0.5). **c-e,** Typical cryo-EM map densities with the fitted atomic model. Neu5Ac: *N*-acetylneuraminic acid, Gal: galactose, GlcNAc: *N*-acetylglucosamine.

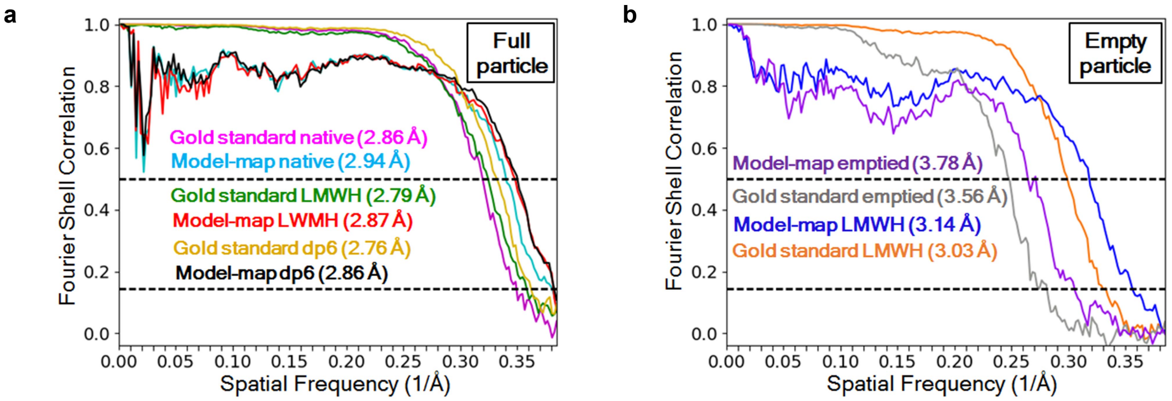

Supplementary Figure 5. Resolution assessment of cryo-EM maps of empty particles (a) and full particles (b) based on FSC curves. The two types of FSC curves are defined as in Supplementary Figure 4.

a

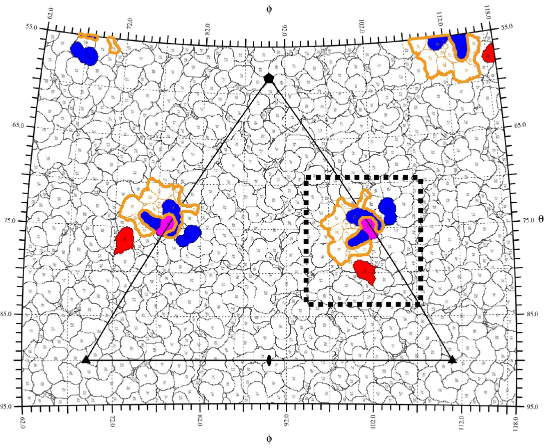

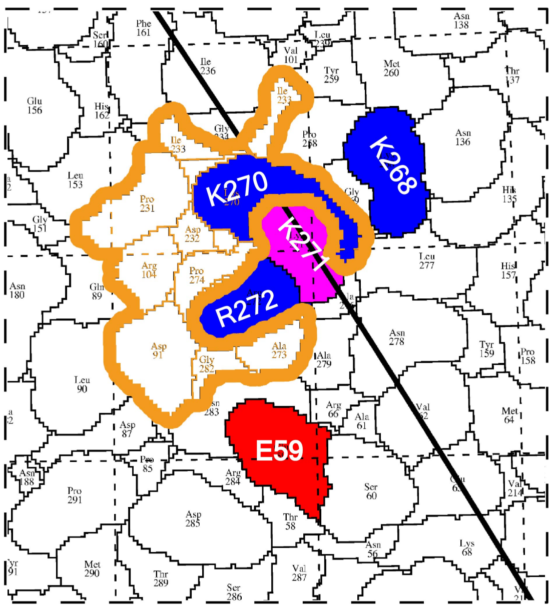

b

	268	270	271	272
Fermon	Lys	Arg	Asp	Thr
2042	Lys	Arg	Glu	Arg
947	Lys	Lys	Lys	Arg

Supplementary Figure 6. Putative sGAG-binding residues in EV-D68-947 are in close proximity to the Sia-binding site on the virus. a, A map of amino acid residues on the outer surface of EV-D68-947. A black dashed rectangle indicates the limit of the close-up view shown on the right. An orange contour outlines the sialic acid binding site. The residues VP1 Lys^{271} (magenta) and VP3 Glu^{59} (red), which allow EV-D68 mutant 2042-4/7 to bind sGAGs, are located near the Sia-binding site. VP1 residues Lys^{268}, Lys^{270}, Lys^{271}, and Arg^{272} (blue and magenta) form a basic patch in EV-D68-947 **b**, Sequence comparisons show that this basic patch is not present in EV-D68-Fermon or EV-D68-2042.

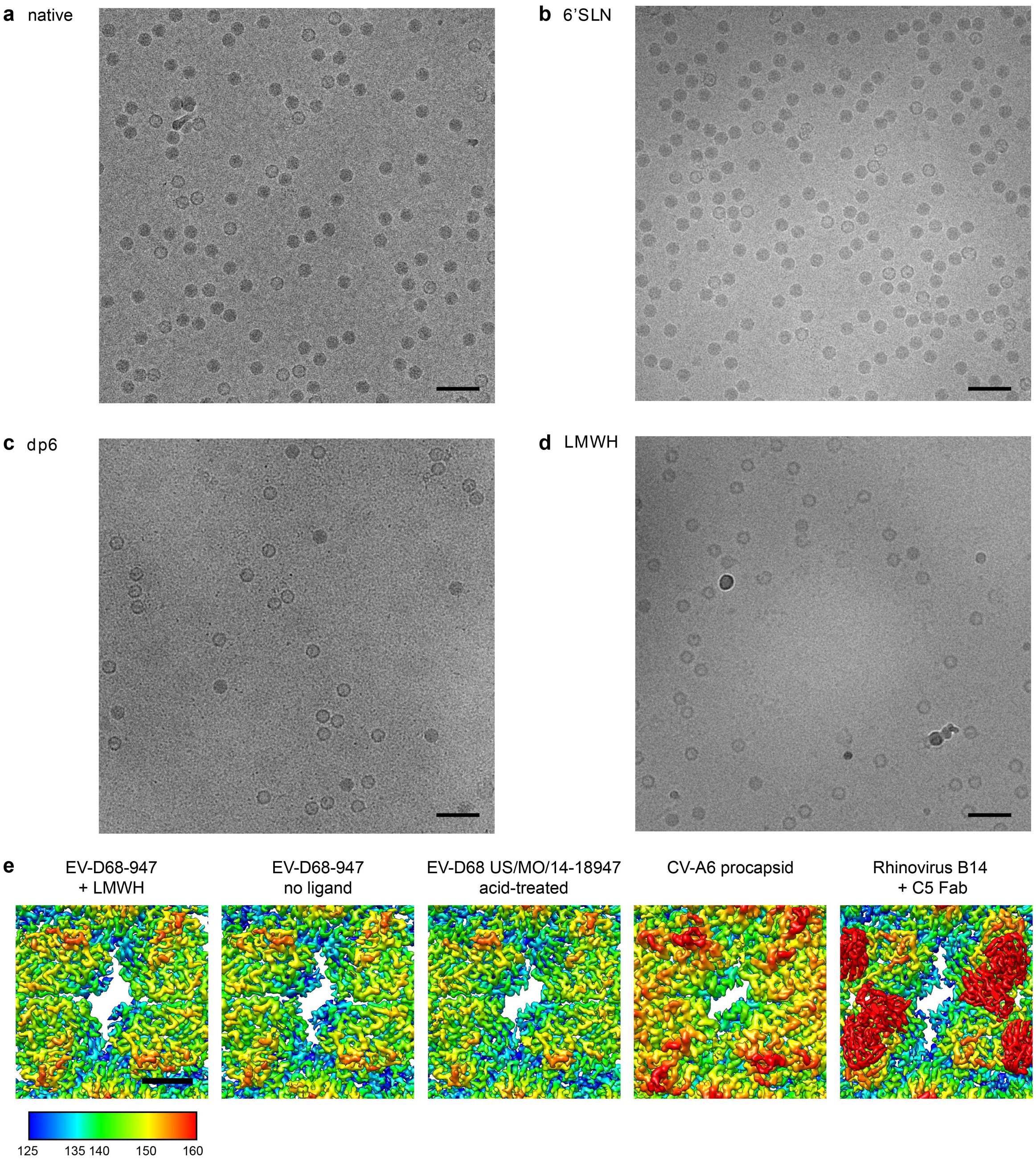

Supplementary Figure 7. Sulfated glycosaminoglycans cause uncoating of EV-D68-947 *in vitro*. a-d, Purified EV-D68-947 was incubated in the absence of a receptor analogue **(a),** with 10 mg mL^{-1} 6'-sialyl-N-acetyllactosamine (6'SLN) **(b)**, with 5 mg mL^{-1} heparin-derived hexasaccharide (dp6) **(c)**, or with 5 mg mL^{-1} low-molecular-weight-heparin (LMWH) **(d)** at 33°C for 1hr. In each panel, a typical portion of a cryo-EM micrograph of EV-D68 particles is shown. Scalebars represent 100 nm. **e**, Close-up views of the openings around the two-fold axis in empty particles of different viruses when looking along an icosahedral two-fold axis The color scale indicates the radial distance to the particle center (Å). The accession numbers for cryo-EM maps shown in this panel are EMD-7634 (EV-D68-947 with LMWH), EMD-7635 (EV-D68-947 no ligand), EMD-7589 (EV-D68 US/MO/14-18947 acid-treated), EMD-6752 (CV-A6 procapsid), and EMD-8763 (rhinovirus B14 in complex with C5 Fab). These maps were all low pass filtered to 3.6 Å resolution. The scalebar represents 35 Å**.**

Supplementary Table 1. Statistics for cryo-EM data collection and processing

	Full-native	Emptied	Full-LMWH	Emptied-LMWH	Full-dp6	Full-6'SLN
**Data collection and processing**						
Microscope	Titan Krios	Titan Krios	Titan Krios	Titan Krios	Titan Krios	Titan Krios
Accelerating voltage (kV)	300	300	300	300	300	300
Camera	K2 Summit	K2 Summit	K2 Summit	K2 Summit	K2 Summit	K2 Summit
No. of micrographs[a]	218	218	872	872	338	330
Pixel size[b] (Å/pixel)	1.30	1.30	1.30	1.30	1.30	1.00
Dose rate (e^-/pixel/s)	8	8	8	8	8	4
Total dose (e^-/Å^2)	33	33	33	33	33	25
Frame rate (ms)	250	250	200	200	200	100
Defocus (µm)	0.5-3.1	0.5-3.1	0.6-4.5	0.6-4.5	0.7-5.4	0.4-3.6
No. particles for reconstruction	4306	2107	5104	9226	6693	5938
Resolution[c] (Å)	2.86	3.56	2.79	3.03	2.76	2.43
Map sharpening B-factor (Å^2)	-111.7	-144.0	-94.1	-118.2	-104.9	-81.9
**Model Statistics**						
Correlation coefficient[d]	0.868	0.836	0.872	0.859	0.862	0.858
No. of atoms						
Protein	6306	5257	6278	5165	6270	6277
Water	0	0	157	0	198	337
Ligand	0	0	0	0	0	46
Avg. B-factor (Å^2)	23.2	84.0	26.6	54.0	24.6	21.2
Avg. B-factor for ligand (Å^2)	-	-	-	-	-	40.5
r.m.s deviations[e]						
Bond lengths (Å)	0.009	0.012	0.010	0.009	0.009	0.011
Bond angles (°)	1.197	1.296	1.236	1.197	1.199	1.308
Ramachadran plot[e]						
Favored (%)	96.8	90.3	95.5	95.9	97.2	95.5
Allowed (%)	3.2	8.8	4.4	4.1	2.8	4.4
Outliers (%)	0.0	0.9	0.1	0.0	0.0	0.1

[a]Micrographs from which particles were selected
[b]Physical pixel size
[c]Estimated base on the Fourier shell correlation between two independently reconstructed half maps (FSC cutoff = 0.143)
[d]Between the cryo-EM map and a map calculated based on the atomic model where the resolution corresponds to that of the cryo-EM map
[e]According to the criteria of MolProbity

Supplementary Table 2. Mutations observed in EV-A71 BrCr after passaging five times in H1-HeLa PLA2G16 knockout cells

**Genomic position**	**Nucleotide**		**Protein**	**Residue**	**Amino acid**	
	Passage 0	Passage 5			Passage 0	Passage 5
1363	G	A	VP2	138	G	D
2318	U	C/U	VP3		Silent mutation	
2985	U	A	VP1	183	L	I
3109	C	U	VP1	224	A	V
3160	T	C	VP1	241	L	S

The genomic positions are numbered according to the EV-A71 strain BrCr-TR complete genome (AB204852.1).

Supplementary Table 3. Oligonucleotide primers used in this study.

**Primer use**	**Sequence (5’-3’)**
Biotinylated primer for LAM-PCR in haploid screen	Double biotin/GGTCTCCAAATCTCGGTGGAAC
Single-stranded DNA linker for LAM-PCR product	Phospho/ATCGTATGCCGTCTTCTGCTTGACTCAGTAGTTGTGCGATGGATTGATG/dideoxycytidine
Forward primer final PCR for Illumina sequencing	CAAGCAGAAGACGGCATACGA
Reverse primer final PCR for Illumina sequencing	AATGATACGGCGACCACCGAGATCTGATGGTTCTCTAGCTTGCC
EV-D68 cDNA synthesis	TTTTTTTTTTTTTTTTTTTTGG
Forward primer for amplification of the EV-D68 947 complete genome and the EV-D68 2042 5’ fragment (*XmaI* site)	TATCCCGGGTTCTTAAAACAGCCTTGGGGT
Reverse primer for amplification of the EV-D68 2042 5’ fragment (*SpeI* site)	GGACAGACTAGTAAACCACCGCA
Forward primer for amplification of the EV-D68 2042 3’ fragment	ATTTGCTTTGAAGGCCCAG
Reverse primer for amplification of the EV-D68 947 complete genome and the EV-D68 2042 3’ fragment (*SalI* site)	CAGGTCGACTTTTTTTTTTTTTTTTTTTTGGCCCCCAAGTGACCAAAAT
Forward primer to introduce EV-D68 947 mutation R116K	AATGCCACAAaATTCCATCAAG
Reverse primer to introduce EV-D68 947 mutation R116K	ACACTGCACATGAATCAAG
Forward primer to introduce EV-D68 947 mutation E59K	TAACAACACAaAAAGTGCGGTTG
Reverse primer to introduce EV-D68 947 mutation E59K	ATCTCCATCATTGATTCCACTTG
Forward primer to introduce EV-D68 947 mutation K169R	ACCCCAGAAAgGCAAGATTCATTC
Reverse primer to introduce EV-D68 947 mutation K169R	AAGAGCACCAGTGGGTAC
Forward primer to introduce EV-D68 947 mutation K270R	TACAAAGGTAgAAAGAGAGCAC
Reverse primer to introduce EV-D68 947 mutation K270R	ATTTGCATTTGCAATGCTC
Forward primer to introduce EV-D68 947 mutation K271E	CAAAGGTAAAgAGAGAGCACC
Reverse primer to introduce EV-D68 947 mutation K271E	TAATTTGCATTTGCAATGC

Restriction sites are underlined; sites of mutations are shown in lowercase.
