# Supplementary material for: Bypassing pan-enterovirus host factor PLA2G16
Source: Nat Commun. 2019 Jul 18;10:3171. doi: 10.1038/s41467-019-11256-z (PMC6639302; doi:10.1038/s41467-019-11256-z)
Supplement: Supplementary file 3 — Reporting Summary [file 41467_2019_11256_MOESM3_ESM.pdf]

nature research

Corresponding author(s): Frank J.M. van Kuppeveld

Last updated by author(s): Jun 17, 2019

Reporting Summary

Nature Research wishes to improve the reproducibility of the work that we publish. This form provides structure for consistency and transparency in reporting. For further information on Nature Research policies, see Authors & Referees and the Editorial Policy Checklist.

Statistics

For all statistical analyses, confirm that the following items are present in the figure legend, table legend, main text, or Methods section.

n/a	Confirmed	
☒	☐	The exact sample size (n) for each experimental group/condition, given as a discrete number and unit of measurement
☒	☐	A statement on whether measurements were taken from distinct samples or whether the same sample was measured repeatedly
☐	☒	The statistical test(s) used AND whether they are one- or two-sided<br>*Only common tests should be described solely by name; describe more complex techniques in the Methods section.*
☒	☐	A description of all covariates tested
☐	☒	A description of any assumptions or corrections, such as tests of normality and adjustment for multiple comparisons
☒	☐	A full description of the statistical parameters including central tendency (e.g. means) or other basic estimates (e.g. regression coefficient) AND variation (e.g. standard deviation) or associated estimates of uncertainty (e.g. confidence intervals)
☒	☐	For null hypothesis testing, the test statistic (e.g. F, t, r) with confidence intervals, effect sizes, degrees of freedom and P value noted<br>*Give P values as exact values whenever suitable.*
☒	☐	For Bayesian analysis, information on the choice of priors and Markov chain Monte Carlo settings
☒	☐	For hierarchical and complex designs, identification of the appropriate level for tests and full reporting of outcomes
☒	☐	Estimates of effect sizes (e.g. Cohen's d, Pearson's r), indicating how they were calculated

Our web collection on statistics for biologists contains articles on many of the points above.

Software and code

Policy information about availability of computer code

Data collection	*Provide a description of all commercial, open source and custom code used to collect the data in this study, specifying the version used OR state that no software was used.*
Data analysis	*Provide a description of all commercial, open source and custom code used to analyse the data in this study, specifying the version used OR state that no software was used.*

For manuscripts utilizing custom algorithms or software that are central to the research but not yet described in published literature, software must be made available to editors/reviewers. We strongly encourage code deposition in a community repository (e.g. GitHub). See the Nature Research guidelines for submitting code & software for further information.

Data

Policy information about availability of data

All manuscripts must include a data availability statement. This statement should provide the following information, where applicable:

- Accession codes, unique identifiers, or web links for publicly available datasets
- A list of figures that have associated raw data
- A description of any restrictions on data availability

The atomic coordinates of full-dp6, full-native, emptied, emptied-LMWH, full-LMWH, and full-6'SLN have been deposited with in the Protein Data Bank withunder the accession codes 6CV1 [http://dx.doi.org/10.2210/pdb6CV1/pdb], 6CV2 [http://dx.doi.org/10.2210/pdb6CV2/pdb], 6CV3 [http://dx.doi.org/10.2210/pdb6CV3/pdb], 6CV4 [http://dx.doi.org/10.2210/pdb6CV4/pdb], 6CV5 [http://dx.doi.org/10.2210/pdb6CV5/pdb], and 6CVB [http://dx.doi.org/10.2210/pdb6CVB/pdb]. The cryo-EM reconstructions of full-dp6, full-native, emptied, emptied-LMWH, full-LMWH, and full-6'SLN have been deposited with the Electron Microscopy Data Bank under the accession codes EMD-7632, EMD-7633, EMD-7634, EMD-7635, EMD-7636, and EMD-7638. All relevant data are available from the authors. A reporting summary for this article is available as a Supplementary Information file. The source data underlying Figs 1a, c, d, 2b, d, 3a-d, 4d, g, h and Supplementary Figs 1a-d, 2d-f, 3a, c are provided as a Source Data file.

Field-specific reporting

Please select the one below that is the best fit for your research. If you are not sure, read the appropriate sections before making your selection.

☒ Life sciences ☐ Behavioural & social sciences ☐ Ecological, evolutionary & environmental sciences

For a reference copy of the document with all sections, see nature.com/documents/nr-reporting-summary-flat.pdf

Life sciences study design

All studies must disclose on these points even when the disclosure is negative.

Sample size	No statistical methods were used to predetermine sample size. Sample sizes were chosen based on the approximate standard deviation generally observed for a given assay in relation to the effect size.
Data exclusions	In a few occasions data were excluded from an experimental dataset. This was done only in case an immunofluoresence image was not completely focused, resulting in a underestimation of the number of infected cells, as measured by automated cell counting.
Replication	For experiments that have been replicated, the number of replicates is indicated in the figure legends. No experiments were included that showed contradicting results upon repetition.
Randomization	The experiments in this study were not randomized.
Blinding	For the acquisition of data presented in Figure 4d, researchers were blinded to group allocation, since this involved visual quantification of images. For all other experiments this was not necessary, either because data were already quantitative or because data acquisition was automated.

Reporting for specific materials, systems and methods

We require information from authors about some types of materials, experimental systems and methods used in many studies. Here, indicate whether each material, system or method listed is relevant to your study. If you are not sure if a list item applies to your research, read the appropriate section before selecting a response.

Materials & experimental systems

n/a	Involved in the study	
☐	☒	Antibodies
☐	☒	Eukaryotic cell lines
☒	☐	Palaeontology
☒	☐	Animals and other organisms
☒	☐	Human research participants
☒	☐	Clinical data

Methods

n/a	Involved in the study	
☒	☐	ChIP-seq
☒	☐	Flow cytometry
☒	☐	MRI-based neuroimaging

Antibodies

Antibodies used	mouse monoclonal anti-dsRNA (J2; English and Scientific Consulting)<br>rabbit polyclonal antiserum against EV-D68 Fermon capsids (obtained from RIVM)
Validation	The manufacturer of the J2 antibody provides a list of over 500 publications that have previously used this antibody.<br>We have tested the reactivity of the EV-D68 Fermon antiserum ourselves, by staining cells infected with many different EV-D68 strains.

Eukaryotic cell lines

Policy information about cell lines

Cell line source(s)	HAP1, HAP1 CMASKO and HAP1 ICAM-5KO cells were obtained from Horizon Discovery Group plc (Cambridge, UK).<br>HAP1 B3GALT6KO, HAP1 SLC35A1KO, H1-HeLa and H1-HeLa PLA2G16KO cells were obtained from Thijn Brummelkamp (Netherlands Cancer institute, Amsterdam).<br>HeLa-R19 cells were obtained from G. Belov (University of Maryland and Virginia-Maryland Regional College of Veterinary Medicine, US).<br>Huh7/Lunet/T7 cells were obtained from Ralf Bartenschlager (Heidelberg University Hospital, Germany).<br>RD (rhabdomyosarcoma) cells were obtained from the European Collection of Cell Cultures (Catalogue No.: 85111502).
Authentication	None of the cell lines used were authenicated.

Mycoplasma contamination	All cell lines tested negative for mycoplasma contamination.
Commonly misidentified lines<br>(See ICLAC register)	No commonly misidentified cell lines were used in this study.
